# A dynamic ribosomal biogenesis response is not required for IGF-1–mediated hypertrophy of human primary myotubes

**DOI:** 10.1096/fj.201700329R

**Published:** 2017-08-03

**Authors:** Hannah Crossland, James A. Timmons, Philip J. Atherton

**Affiliations:** * Division of Genetics and Molecular Medicine, Guy’s Hospital, King’s College London, London, United Kingdom; and; † School of Medicine, Royal Derby Hospital, University of Nottingham, Derby, United Kingdom

**Keywords:** RNA, skeletal muscle, mTOR

## Abstract

Increased ribosomal DNA transcription has been proposed to limit muscle protein synthesis, making ribosome biogenesis central to skeletal muscle hypertrophy. We examined the relationship between ribosomal RNA (rRNA) production and IGF-1–mediated myotube hypertrophy *in vitro*. Primary skeletal myotubes were treated with IGF-1 (50 ng/ml) with or without 0.5 µM CX-5461 (CX), an inhibitor of RNA polymerase I. Myotube diameter, total protein, and RNA and DNA levels were measured along with markers of RNA polymerase I regulatory factors and regulators of protein synthesis. CX treatment reduced 45S pre-rRNA expression (−64 ± 5% *vs.* IGF-1; *P* < 0.001) and total RNA content (−16 ± 2% *vs.* IGF-1; *P* < 0.001) in IGF-1-treated myotubes. IGF-1-mediated increases in myotube diameter (1.27 ± 0.09-fold, *P* < 0.05 *vs.* control) and total protein (+20 ± 2%; *P* < 0.001 *vs.* control) were not prevented by CX treatment. Suppression of rRNA synthesis during IGF-1 treatment did not prevent early increases in AKT (+203 ± 39% *vs.* CX; *P* < 0.001) and p70 S6K1 (269 ± 41% *vs.* CX; *P* < 0.001) phosphorylation. Despite robust inhibition of the dynamic ribosomal biogenesis response to IGF-1, myotube diameter and protein accretion were sustained. Thus, while ribosome biogenesis represents a potential site for the regulation of skeletal muscle protein synthesis and muscle mass, it does not appear to be a prerequisite for IGF-1-induced myotube hypertrophy *in vitro.*—Crossland, H., Timmons, J. A., Atherton, P. J. A dynamic ribosomal biogenesis response is not required for IGF-1–mediated hypertrophy of human primary myotubes.

Ribosomal biogenesis is a coordinated, multistep process that plays a central biochemical role in cellular translational capacity and protein synthesis (1–3). Transcription of 45S pre–ribosomal DNA (rDNA) by RNA polymerase I (Pol I) is considered a rate-limiting step in ribosomal biogenesis, which is in turn regulated by a number of key factors, including upstream binding factor (UBF) and the selectivity factor 1 (SL1) complex, which are recruited at the rDNA promoter (2). These events result in the activation of transcription initiation factor 1A (TIF1A), which interacts with Pol I and enables initiation of Pol I–dependent synthesis of 45S ribosomal RNA (rRNA). The pre-rRNA 45S transcript is subsequently processed to generate 3 mature rRNA subunits—28S, 18S, and 5.8S—which assemble to form the mature ribosome, along with a fourth rRNA transcribed by RNA Pol III (5S) and ∼80 ribosomal proteins (1).

Two of the major regulators considered important for ribosomal biogenesis are the mammalian target of rapamycin (mTOR) C1 and c-Myc signaling pathways (3, 4). Inhibition of mTORC1 by rapamycin was shown to inactivate TIF1A and UBF (5, 6), indicating that mTORC1 plays a role in regulation of Pol I transcription. c-Myc has been demonstrated to regulate UBF (7), as well as to directly regulate Pol I–dependent rRNA transcription through binding to the promoter of rDNA (8), thus transcribing a number of ribosomal protein genes (9). c-Myc, initially described as a oncogene, has multiple actions, including enhancing protein synthesis through transcription of ribosomal protein RNA (9); it is regulated both transcriptionally and posttranscriptionally in muscle cells in response to growth factors (10). Further, c-Myc has been reported to increase after resistance exercise in animal models and humans (11, 12), and c-Myc may act as a regulator of the Pol I machinery (13) during human skeletal muscle hypertrophy.

Increased muscle protein synthesis (MPS) after resistance exercise could be driven through increases in translational efficiency (*i.e.*, the rate of protein synthesis per ribosome). Alternatively, resistance exercise could also increase translational capacity (*i.e.*, the amount of translational machinery per unit of cells per tissue), suggesting that ribosome number would then be a key determinant of translational capacity. Indeed, several studies have reported increases in markers of ribosomal biogenesis during muscle hypertrophy (11, 14–16). These observations imply that ribosomal biogenesis may represent a central point for regulating MPS and muscle mass, and this has led to the hypothesis that *de novo* ribosome biogenesis may therefore be a prerequisite for skeletal muscle growth (17).

Whether *de novo* ribosome biogenesis is obligatory for skeletal muscle growth or whether ribosome number defines a maximum potential capacity for translation in skeletal muscle is less clear. Studies have reported that *in vitro*, serum-induced myotube hypertrophy (*i.e.*, a complex mix of growth factors, hormones, and nutrients) was inhibited when rRNA induction was blocked using a chemical inhibitor of Pol I (14), and that in C2C12 myotubes, increases in protein content and MPS with high serum stimulation were prevented by Pol I inhibition (18). These findings support an essential role for ribosomal number in muscle cellular growth when using this murine immortalized cell-line model of muscle biology, but whether this holds true in human primary muscle cells is not known. Therefore, the aim of the present study was: *1*) to explore the role of ribosomal biogenesis in human primary myocyte size regulation *in vitro*, *2*) to determine whether ribosomal biogenesis is required for skeletal muscle hypertrophy in response to an established *in vivo* trophic signal (IGF-1) (19–21), and *3*) to identify the signaling mediators driving these changes. We present a comprehensive analysis of the impact of inhibition of ribosome biogenesis using a specific chemical inhibitor of RNA, Pol I, on factors linked to regulation of rDNA transcription and protein turnover during hypertrophy.

## MATERIALS AND METHODS

### Primary cell culture and drug treatments

Human satellite cells were isolated from muscle biopsy samples from healthy young adult donors (2 men and 1 woman), as previously described (22). This procedure was approved by the ethics committee of the Karolinska Institutet (Stockholm, Sweden). Briefly, muscle biopsy samples were digested by incubating in 5 ml TrypLE Express (Thermo Fisher Scientific, Waltham, MA, USA) at 37°C and 5% CO_2_, with stirring, for 20 min. Digestion was repeated twice, and isolated cells were recovered and maintained in growth medium [DMEM/Nutrient Mixture F-12 (DMEM/F-12); Thermo Fisher Scientific] containing 20% (v/v) fetal bovine serum (Sigma-Aldrich, St. Louis, MO, USA), 1% (v/v) antibiotic–antimycotic solution, and 4 mM l-glutamine (Thermo Fisher Scientific). Enrichment of myogenic cells was achieved using magnetic-activated cell sorting using anti-CD56 microbeads, as previously described (22). Myoblast purity (assessed through desmin immunofluorescent staining) was above 90% for all 3 donors. Magnetic-activated cell–sorted myogenic precursor cells were cultured on collagen-coated culture dishes in growth medium. Cells were maintained at 37°C with 5% CO_2_, and myoblasts were used for experimentation at passages 5 to 6.

When cells reached ∼90% confluence, differentiation was initiated by changing the medium to DMEM/F-12 containing 2% (v/v) horse serum (Sigma-Aldrich), with 4 mM l-glutamine and 1% (v/v) antibiotic–antimycotic solution (Thermo Fisher Scientific). Six days after the induction of differentiation, the medium was changed, and after 24 h, cells were treated with IGF-1 (50 ng/ml) with or without 0.5 µM CX-5461 (CX; Cayman Chemicals, Ann Arbor, MI, USA). All cells were treated with 50 µM cytosine arabinoside (Sigma-Aldrich) to reduce proliferation of nondifferentiated myoblasts. The dose of CX was chosen on the basis of preliminary experiments, whereby doses at 1 µM and above caused significant declines in total DNA after 24 h, while doses below 0.25 µM did not reduce total RNA content after 24 h (data not shown).

Samples were collected after 4 or 24 h of treatment. Samples were collected in Trizol (Thermo Fisher Scientific) for RNA isolation, homogenization buffer (50 mM Tris-HCl, pH 7.5, 1 mM EDTA, 1 mM EGTA, 10 mM β-glycerophosphate, 50 mM NaF), and complete protease inhibitor cocktail tablet (Roche, Basel, Switzerland) for protein extraction, or 0.3 M NaOH for total protein, RNA, and DNA extraction and quantification. Independent experiments were carried out for each donor (*n* = 3–4 well replicates for each treatment group). Light microscope images of myotubes were also taken after experimentation for measurement of myotube diameter. Mean diameter was calculated from measurement of 200 myotubes across 10 images for each treatment.

### Immunofluorescent staining

After experimentation, cells were washed in PBS and fixed using ice-cold 1:1 acetone:methanol. After fixation, cells were washed in PBS and incubated in 5% (v/v) goat serum for 30 min at room temperature. Cells were then incubated in rabbit anti-desmin monoclonal antibody (Abcam, Cambridge, MA, USA) for 1 h at room temperature, and after further washing were incubated with anti-rabbit FITC-conjugated secondary antibody (Abcam). After washing with PBS, cells were mounted and stained with DAPI using Fluoroshield mounting medium with DAPI. Images were used for assessment of nuclei per myotube.

### Total protein, RNA, and DNA measurements

After experimentation, cells were collected in 0.3 M NaOH, and samples were incubated at 37°C for 20 min for extraction of total alkaline-soluble protein. After protein quantification, 1 M perchloric acid was added to the samples, and samples were left at 4°C for 30 min. After centrifugation, the supernatant was quantified for RNA. To the pellet, 2 M perchloric acid was added, and samples were incubated at 70°C for 1 h. The supernatant was used for total DNA quantification.

### RNA extraction, cDNA synthesis, and real-time PCR

RNA was isolated using Trizol reagent (Thermo Fisher Scientific) according to the manufacturer’s instructions. After extraction, RNA was resuspended in 20 µl of RNase-free water, and quantity and quality were determined by a NanoDrop 2000 (Thermo Fisher Scientific). cDNA was synthesized by reverse transcription using the High Capacity cDNA synthesis kit (Thermo Fisher Scientific) with 500 ng RNA. Samples were diluted 1:10 with RNase-free water after cDNA synthesis, and real-time PCR was performed using 1 µl cDNA in duplicate and 6 µl master mix containing SYBR Select Master Mix (Thermo Fisher Scientific) and primers targeting the following genes: 45S pre-rRNA (designed to target the 5′ external transcribed spacer), 28S rRNA, 5.8S rRNA (designed to span the 5.8S and 5′ internal transcribed spacer region), polymerase 1 subunit A/B/C/E (POLR1A/B/C/E), UBF, TIF1A, c-MYC, TATA-box binding protein associated factor, RNA polymerase I, A (TAF1A), ribosomal protein (RP) L13A, RPL32, RPS5, and RPS19 (Supplemental Table 1). Samples were analyzed using a Viia 7 real-time PCR machine (Thermo Fisher Scientific) with the following thermal cycling conditions: 2 min at 50°C, 10 min at 95°C, and 40 cycles of 15 s at 95°C and 1 min at 60°C. 18S rRNA was used for normalization because it did not differ between treatment groups. The Δ*C_t_* method (23) was used to calculate relative changes in target mRNA abundance.

### Protein extraction and Western blot analysis

Cell protein extracts were prepared by repeatedly passing samples through gel-loading pipette tips. Samples were centrifuged at 13,000 *g* for 10 min at 4°C, and lysates (5 µg protein) were loaded onto Criterion XT 12% Bis-Tris gels (Bio-Rad, Hercules, CA, USA) at 200 V for 1 h. Samples were transferred to PVDF membrane at 100 V for 1 h, and membranes were blocked using 2.5% (w/v) bovine serum albumin for 1 h at room temperature. After this, membranes were incubated overnight at 4°C with the following primary antibodies (all diluted 1:2000): phosphorylated mTOR Ser2448 (5536), total mTOR (2983), phosphorylated p70 S6K1 Thr389 (9234), total p70 S6K1 (9202), phosphorylated AKT Ser473 (4060), total AKT (4685), phosphorylated 4E-BP1 Thr37/46 (2855), total 4E-BP1 (9452), total c-Myc (13987), phosphorylated retinoblastoma (Rb) Ser780 (3590), total TIF1A (ab57994), phosphorylated UBF Ser484 (ab182583), total UBF (ab75781), phosphorylated forkhead box O 3a (FOXO3a) Ser253 (13129), muscle RING finger 1 (MuRF1) (MP3401), light chain 3B (LC3B; 2775), and ubiquitin (3933). All antibodies were purchased from Cell Signaling Technology (Danvers, MA, USA) except for TIF1A, phospho-UBF, and total UBF, which were purchased from Abcam, and MuRF1, which was purchased from ECM Biosciences (Versailles, KY, USA). After incubation with primary antibody, membranes were washed for 3 times 5 min with Tris-buffered saline–Tween, then incubated for 1 h at room temperature with horseradish peroxidase–conjugated secondary antibody (all anti-rabbit except for TIF1A, which was anti-mouse; New England Biolabs, Ipswich, MA, USA), diluted 1:2000. Bands were visualized using enhanced chemiluminescence detection reagent (EMD Millipore, Billerica, MA, USA) and a Chemidoc XRS imaging system (Bio-Rad). Coomassie Brilliant Blue staining of the membrane was used for normalization against total protein loading.

### Statistical analyses

Data were analyzed by 1-way ANOVA, with Tukey’s multiple comparison test used to examine differences between treatment groups. For the real-time PCR data, 2-way ANOVA with Tukey’s multiple comparison test was used to examine differences between groups. All data are presented as means ± sem, with *P* < 0.05 being considered statistically significant. All data were analyzed using GraphPad Prism v.6 (La Jolla, CA, USA).

## RESULTS

### Myotube diameter and nuclei per myotube

**Figure 1.**
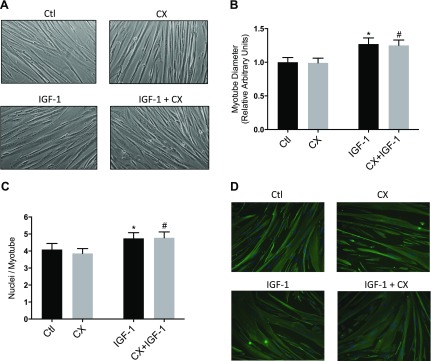
Images of human myotubes (*A*), myotube diameter measurements (*B*), nuclei per myotube (*C*), and desmin staining of myotubes (*D*) after 24 h of IGF-1 with or without CX treatment. Light microscope images were taken from differentiated myotubes after 24 h of 50 ng/ml IGF-1 treatment with or without 0.5 µM CX. Human primary myotubes were stained by immunofluorescence for desmin after incubation for 24 h with IGF-1 and/or CX. Number of nuclei per myotube was calculated for each group.

Treatment of differentiated myotubes for 24 h with CX did not affect myotube diameter (**Fig. 1*A, B***). IGF-1 caused an increase in myotube diameter (1.27 ± 0.09-fold, *P* < 0.05 *vs.* control), and in the presence of CX and IGF-1, myotube diameter was higher than with CX alone (1.25 ± 0.08-fold, *P* < 0.05 *vs.* CX; Fig. 1*A, B*). The number of nuclei per myotube (assessed by desmin immunofluorescence) was no different between CX-treated and untreated cells (Fig. 1*C, D*), but with IGF-1 there was an increase in the number of nuclei per myotube after 24 h (4.7 ± 0.34 with IGF-1, 4.1 ± 0.35 with control; *P* < 0.05 *vs.* control). CX did not prevent the IGF-1-induced increase in number of nuclei per myotube (4.7 ± 0.35 with CX + IGF-1; *P* < 0.05 *vs.* CX; Fig. 1*C, D*).

### Pol I specificity and total protein, RNA, and DNA measurements

**Figure 2.**
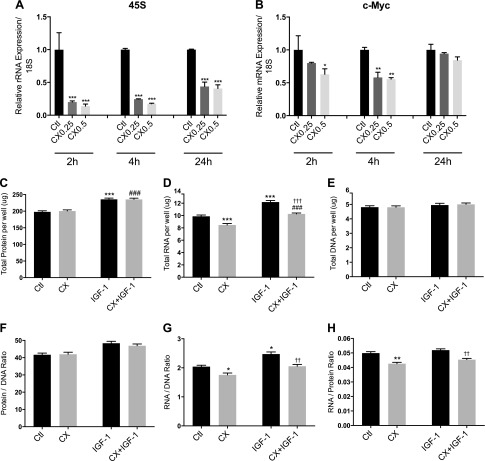
Dose–time course of CX treatment and total protein, RNA, and DNA content in human primary myotubes after IGF-1 treatment with or without CX. *A*, *B*) Expression of 45S rRNA (*A*) and c-Myc (*B*) mRNA was measured in human primary myotubes after 2, 4, and 24 h of CX treatment (0.25 or 0.5 µM). *C*–*E*) Total protein (*C*), RNA (*D*), and DNA (*E*) were measured in human primary myotubes after 24 h of IGF-1 (50 ng/ml) treatment with or without 0.5 µM CX. *F*–*H*) From these values, the protein/DNA (*F*), RNA/DNA (*G*) and RNA/protein (*H*) ratios were calculated. Data are expressed as means ± sem from 3 independent experiments (*n* = 4–6 replicates per treatment). *P < 0.05, **P < 0.01, ***P < 0.001 *vs.* untreated control; ^###^*P* < 0.001 *vs.* CX, ^††^*P* < 0.01, ^†††^*P* < 0.001 *vs.* IGF-1.

A dose–time course of CX treatment in myotubes was undertaken to verify specificity of the compound for RNA Pol I *vs.* RNA Pol II. At 2, 4, and 24 h, CX caused a reduction in 45S rRNA expression relative to untreated controls (for 0.25 µM: −80 ± 2% *vs.* control at 2 h; *P* < 0.001, −75 ± 1% *vs.* control at 4 h; *P* < 0.001, −56 ± 7% *vs.* control at 24 h; *P* < 0.001, for 0.5 µM: −86 ± 3% *vs.* control at 2 h; *P* < 0.001, −83 ± 1% *vs.* control at 4 h; *P* < 0.001, −59 ± 6% *vs.* control at 24 h; *P* < 0.001; **Fig. 2*A***). Expression of c-Myc (used to assess Pol II transcription) was altered by CX treatment with 0.5 µM at 2 h (−37 ± 8% *vs.* control; *P* < 0.05) and both doses at 4 h (−42 ± 8% *vs.* control; *P* < 0.01 at 0.25 µM, 44 ± 2% *vs.* control; *P* < 0.01 at 0.5 µM; Fig. 2*B*). Nevertheless, a greater preference for inhibition of RNA Pol I than RNA Pol II was observed with CX treatment. Total protein and DNA per well was not altered by CX alone (Fig. 2*C, E*), but after 24 h, total RNA (−15 ± 2% *vs.* control; *P* < 0.001; Fig. 2*D*) and the RNA/DNA ratio (−14 ± 3% *vs.* control; *P* < 0.05) were lower. CX also caused a reduction in the RNA/protein ratio at 24 h (−15 ± 2% *vs.* control; *P* < 0.01; Fig. 2*H*). IGF-1 treatment for 24 h resulted in higher total protein (+20 ± 2%; *P* < 0.001 *vs.* control; Fig. 2*C*), total RNA (+23 ± 3%; *P* < 0.001 *vs.* control; Fig. 2*D*), and RNA/DNA ratio (+21 ± 4%; *P* < 0.05 *vs.* control; Fig. 2*G*). There was no change in total DNA or the protein/DNA ratio with IGF-1 treatment (Fig. 2*E, F*). The increase in total RNA and RNA/DNA ratio with IGF-1 was lower with CX treatment (*P* < 0.001 *vs.* IGF-1; Fig. 2*B* and *P* < 0.01 *vs.* IGF-1; Fig. 2*G*), but the increase in total protein with IGF-1 was not prevented by the addition of CX (Fig. 2*C*).

### mRNA expression

**Figure 3.**
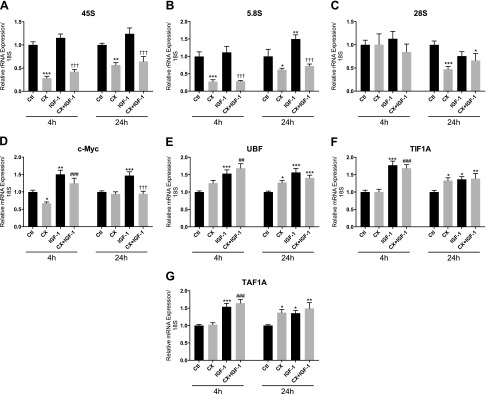
Expression of rRNA and Pol I regulatory factors after IGF-1 treatment with or without CX. Expression of 45S (*A*), 5.8S (*B*), 28S (*C*), c-Myc (*D*), UBF (*E*), TIF1A (*F*), and TAF1A (*G*) after 4 and 24 h of IGF-1 treatment (50 ng/ml) with or without 0.5 µM CX in human primary myotubes. Data are normalized to 18S expression and are expressed as means ± sem from 3 independent experiments (*n* = 4 replicates per treatment). *P < 0.05, **P < 0.01; ***P < 0.001 *vs.* untreated control; ^##^*P* < 0.01, ^###^*P* < 0.001 *vs.* CX; ^†††^*P* < 0.001 *vs.* IGF-1.

Expression of 45S rRNA was significantly reduced by CX treatment both with and without IGF-1 after 4 h (−72 ± 4% *vs.* control; *P* < 0.001 with CX, −64 ± 5% *vs.* IGF-1; *P* < 0.001 with CX + IGF-1) and 24 h (−44 ± 5% *vs.* control; *P* < 0.01 with CX, −48 ± 11% *vs.* IGF-1; *P* < 0.001 with CX + IGF-1; **Fig. 3*A***), and 5.8S expression showed a similar pattern of changes (Fig. 3*B*). Expression of 28S was unaffected by treatments except for at 24 h, where CX treatment with or without IGF-1 caused a reduction in expression of 28S relative to untreated controls (−53 ± 6% *vs.* control; *P* < 0.001 with CX, −34 ± 15% *vs.* control; *P* < 0.05 with CX + IGF-1; Fig. 3*C*).

Expression of c-Myc mRNA was lower with CX at 4 h (−33 ± 4% *vs.* control; *P* < 0.05; Fig. 3*D*), but it returned to baseline levels by 24 h. IGF-1 caused an increase in c-Myc mRNA at both time points (+51 ± 12% *vs.* control at 4 h; *P* < 0.01; +47 ± 11% *vs.* control at 24 h; *P* < 0.001; Fig. 3*D*). Addition of CX to IGF-1-treated cells did not affect c-Myc expression compared to IGF-1 alone at 4 h, but its expression was significantly lower compared to the IGF-1 group at 24 h (*P* < 0.001 *vs.* IGF-1; Fig. 3*D*). UBF mRNA was increased by CX treatment at 24 h (+28 ± 6% *vs.* control; *P* < 0.05), and IGF-1 alone increased UBF mRNA at both time points (+53 ± 11% *vs.* control at 4 h; *P* < 0.001; +56 ± 11% *vs.* control at 24 h; *P* < 0.001; Fig. 3*E*). Addition of CX to IGF-1-treated cells did not prevent this increase at either time point, although at 24 h, there was no difference in UBF expression between the CX and CX + IGF-1 group, but mRNA levels were elevated above control (+41 ± 8% *vs.* control; *P* < 0.001; Fig. 3*E*). Expression of TIF1A mRNA was higher with CX at 24 h (+33 ± 8% *vs.* control; *P* < 0.05; Fig. 3*F*). IGF-1 caused an increase in TIF1A mRNA at both time points (+77 ± 12% *vs.* control at 4 h; *P* < 0.001; +37 ± 8% *vs.* control at 24 h; *P* < 0.05; Fig. 3*F*), and this increase was not affected by the addition of CX at either time point. Similar patterns of change in mRNA expression across treatments were observed for TAF1A (Fig. 3*G*).

**Figure 4.**
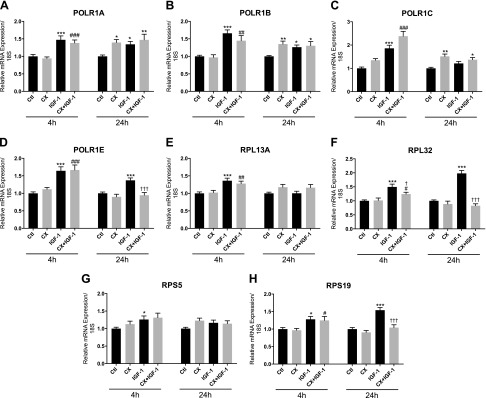
mRNA expression of RNA Pol I subunits and ribosomal protein genes after IGF-1 treatment with or without CX. Expression of POLR1A (*A*), POLR1B (*B*), POLR1C (*C*), POLR1E (*D*), RPL13A (*E*), RPL32 (*F*), RPS5 (*G*), and RPS19 (*H*) mRNA after 4 and 24 h of IGF-1 treatment (50 ng/ml) with or without 0.5 µM CX in human primary myotubes. Data are normalized to 18S expression and expressed as means ± sem from 3 independent experiments (*n* = 4 replicates per treatment). *P < 0.05, **P < 0.01, ***P < 0.001 *vs.* untreated control; ^#^*P* < 0.05, ^##^*P* < 0.01, ^###^*P* < 0.001 *vs.* CX; ^†^*P* < 0.05, ^†††^*P* < 0.001 *vs.* IGF-1.

Expression of RNA Pol I subunit POLR1A was higher with CX treatment after 24 h (+39 ± 9% *vs.* control; *P* < 0.05; **Fig. 4*A***), and with IGF-1 at 4 h (+47 ± 11% *vs.* control; *P* < 0.001) and 24 h (+35 ± 8% *vs.* control; *P* < 0.001; Fig. 4*A*). When CX was added to IGF-1-treated cells, expression of POLR1A was similar to IGF-1 alone (+41 ± 8% *vs.* CX at 4 h; *P* < 0.001, +47 ± 16% *vs.* control at 24 h; *P* < 0.01; Fig. 4*A*). Similar patterns in expression changes were observed for POLR1B (Fig. 4*B*), POLR1C (Fig. 4*C*), and POLR1E (Fig. 4*D*), except that for POLR1E, at 24 h, there was no change in expression after CX or CX + IGF-1 treatment relative to the control group. Ribosomal protein gene RPL13A mRNA was unaffected across groups except at 4 h, where RPL13A was higher with IGF-1 and CX + IGF-1 (+36 ± 7% *vs.* control; *P* < 0.001; +27 ± 7% *vs.* CX; *P* < 0.01, respectively; Fig. 4*E*). RPL32 expression was unaffected by CX alone but was elevated by IGF-1 at 4 h (+50 ± 10% *vs.* control; *P* < 0.001) and 24 h (+98 ± 10% *vs.* control; *P* < 0.001; Fig. 4*F*). With CX + IGF-1, RPL32 expression was increased relative to the CX group at 4 h (+22 ± 6% *vs.* CX; *P* < 0.05) but was significantly lower compared to IGF-1 alone, and at 24 h, expression levels were significantly lower compared to IGF-1 (Fig. 4*F*). Expression of RPS5 was unchanged across treatments at either time point, except for at 4 h, where RPS5 mRNA was increased by IGF-1 (+26 ± 10% *vs.* control; *P* < 0.05; Fig. 4*G*). RPS19 was unaffected by CX alone at either time point but was increased by IGF-1 at 4 h (+28 ± 8% *vs.* control; *P* < 0.05) and 24 h (+54 ± 8% *vs.* control; *P* < 0.001; Fig. 4*H*). Addition of CX to IGF-1-treated cells did not prevent the increase in RPS19 at 4 h, but at 24 h, the increase observed with IGF-1 alone was completely prevented by CX (*P* < 0.001 *vs.* IGF-1; Fig. 4*H*).

### Protein expression and phosphorylation

**Figure 5.**
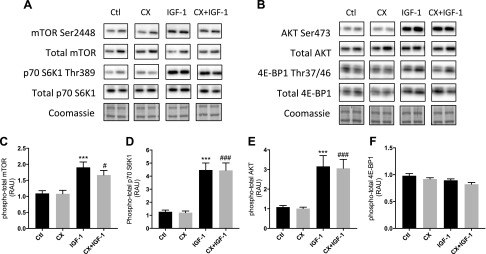
Protein expression and phosphorylation of selected mTOR signaling components in human primary myotubes after IGF-1 treatment with or without CX. *A*, *B*) Representative blots from Western blot analysis of selected signaling targets taken from differentiated myotubes after 4 h of IGF-1 treatment with or without CX. *C*–*F*) Relative changes in phosphorylated (phospho)-total mTOR (*C*), phospho-total p70 S6K1 (*D*), phospho-total AKT (*E*), and phospho-total 4E-BP1 (*F*) after IGF-1 treatment with or without CX. RAU, relative arbitrary units. Data are presented as means ± sem from 3 independent experiments (*n* = 3 per treatment). *P < 0.05, **P < 0.01, ***P < 0.001 *vs.* control; ^#^*P* < 0.05, ^###^*P* < 0.001 *vs.* CX.

Acute changes in anabolic-related signaling proteins were measured after 4 h of IGF-1 treatment with and without CX. Treatment of myotubes with CX alone did not affect mTOR Ser2448 phosphorylation or total mTOR protein at 4 h, and so there was no change in phospho-total mTOR (**Fig. 5*A, C***). With IGF-1 treatment, mTOR Ser2448 phosphorylation, normalized to total mTOR (which was unchanged across all treatments; data not shown), was higher relative to untreated controls (+74 ± 16% *vs.* control; *P* < 0.001; Fig. 5*A, C*). Phospho-total mTOR with CX + IGF-1 treatment was elevated above CX alone (+54 ± 14% *vs.* CX; *P* < 0.05) but was no different from the IGF-1 group (Fig. 5*A, C*). Expression of phosphorylated p70 S6K1 Thr389 and total p70 S6K1 was unaffected by CX treatment at 4 h (Fig. 5*A, D*), whereas IGF-1 treatment increased phospho-total p70 S6K1 (+252 ± 60% *vs.* control; *P* < 0.001; Fig. 5*A, D*). Addition of CX to IGF-1-treated cells did not affect the increase in p70 S6K1 phosphorylation (Fig. 5*A, D*). Treatment with CX did not affect phosphorylated (Ser473) or total AKT (Fig. 5*B, E*), but after 4 h of IGF-1 treatment, phospho-total AKT was higher relative to control (+191 ± 44% *vs.* control; *P* < 0.001; Fig. 5*B, E*). Addition of CX to IGF-1-treated cells did not prevent this increase (Fig. 5*B, E*; +203 ± 39% *vs.* CX; *P* < 0.001). Total AKT was unaffected across treatments (data not shown). Phosphorylated (Thr37/46) or total 4E-BP1 were unaffected by either CX alone, IGF-1, or CX + IGF-1 treatment (Fig. 5*B, F*).

**Figure 6.**
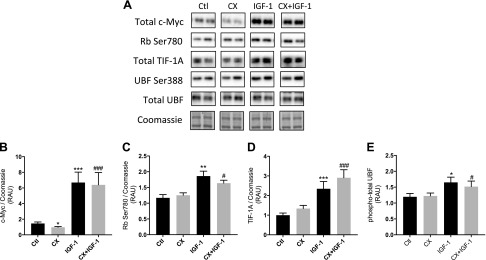
Protein expression and phosphorylation of selected regulators of ribosome biogenesis in human primary myotubes after IGF-1 treatment with or without CX. *A*) Representative blots from Western blot analysis of selected signaling targets taken from differentiated myotubes after 4 h of IGF-1 treatment with or without CX. *B*–*E*) Relative changes in total c-Myc (*B*), phosphorylated Rb Ser780 (*C*), total TIF1A (*D*), and phospho-total UBF (*E*) after IGF-1 treatment with or without CX. RAU, relative arbitrary units. Data are presented as means ± sem from 3 independent experiments (*n* = 3 per treatment). *P < 0.05, ***P < 0.001 *vs.* control; ^#^*P* < 0.05, ^###^*P* < 0.001 *vs.* CX.

Protein expression and/or phosphorylation of selected regulators of ribosome biogenesis were assessed after IGF-1 and CX treatments. Expression of total c-Myc was lower with CX treatment (−33 ± 9% *vs.* control; *P* < 0.05; **Fig. 6*A, B***), whereas IGF-1 caused an increase in total c-Myc (+359 ± 133% *vs.* control; *P* < 0.001). Addition of CX to IGF-1-treated cells did not prevent the increase in c-Myc with IGF-1 (+557 ± 140% *vs.* CX; *P* < 0.001; Fig. 6*A, B*). Phosphorylation of Rb (Ser780) was unchanged with CX treatment but higher with IGF-1 (+59 ± 15% *vs.* control at 4 h; *P* < 0.01; Fig. 6*A, C*). CX did not prevent the increase in Rb phosphorylation in IGF-1-treated cells (+30 ± 9% *vs.* CX; *P* < 0.05). Total TIF1A protein was not affected by CX treatment, while IGF-1 increased TIF1A protein at 4 h (+140 ± 34% *vs.* control; *P* < 0.001; Fig. 6*A, D*). TIF1A protein was significantly higher after CX + IGF-1 treatment (+186 ± 43% *vs.* CX; *P* < 0.001). CX had no effect on expression of phosphorylated (Ser388) or total UBF (Fig. 6*A, E*), whereas phospho-total UBF was higher with IGF-1 treatment (+38 ± 12% *vs.* control; *P* < 0.05). This increase was not prevented by addition of CX (+24 ± 12% *vs.* CX; *P* < 0.05).

**Figure 7.**
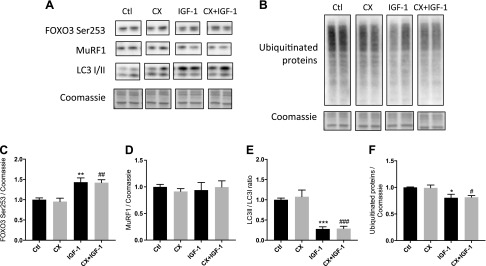
Protein expression and phosphorylation of selected regulators of protein breakdown in human primary myotubes after IGF-1 treatment with or without CX. *A*, *B*) Representative blots from Western blot analysis of selected signaling targets taken from differentiated myotubes after 4 h of IGF-1 treatment with or without CX. *C*–*F*) Relative changes in phosphorylated FOXO3 Ser253 (*C*), total MuRF1 (*D*), LC3II/LC3I ratio (*E*), and ubiquitinated proteins (*F*) after IGF-1 treatment with or without CX. RAU, relative arbitrary units. Data are presented as means ± sem from 3 independent experiments (*n* = 3 per treatment). *P < 0.05, **P < 0.01, ***P < 0.001 *vs.* control; ^#^*P* < 0.05, ^##^*P* < 0.01, ^###^*P* < 0.001 *vs.* CX.

Markers of muscle protein breakdown were measured to assess whether IGF-1-mediated changes in protein degradation were impacted by the inhibition of ribosome biogenesis. Phosphorylation of FOXO3a (Ser253) was unchanged with CX alone but was increased by IGF-1 (+43 ± 9% *vs.* control; *P* < 0.01) and CX + IGF-1 (+49 ± 7% *vs.* CX; *P* < 0.01; **Fig. 7*A, C***). Total MuRF1 protein was unaltered by CX or IGF-1 treatments (Fig. 7*A, D*). Autophagy marker LC3B, presented as the ratio of LC3II/I, was lower with IGF-1 (−72 ± 4% *vs.* control; *P* < 0.001; Fig. 7*A, E*), and this was not altered by the addition of CX to IGF-1-treated cells (−73 ± 4% *vs.* CX; *P* < 0.001; Fig. 7*A, E*). Finally, levels of ubiquitinated proteins, determined by measuring intensity of the entire lane, were unaffected by CX (Fig. 7*B, F*) but were lower with 4 h of IGF-1 (−20 ± 5% *vs.* control; *P* < 0.05; Fig. 7*B, F*) and CX + IGF-1 treatment (−18 ± 2% *vs.* control; *P* < 0.05; Fig. 7*B, F*).

## DISCUSSION

A growing number of studies have provided various levels of evidence that rDNA transcription represents a crucial event before successful muscle hypertrophy, indicating that *de novo* ribosome biogenesis may be necessary for muscle growth (11, 13, 14, 16, 17). For example, in mice, increases in muscle rRNA content, rDNA transcription, and expression of Pol I–related genes were observed at the onset of hypertrophy after synergist ablation (13). Furthermore, in a rodent synergist ablation model, where partial removal of synergist muscles allowed different magnitudes of compensatory hypertrophy, increases in rRNA content showed a strong correlation with relative muscle weight after overload (16). However, there are several methodologic considerations that must be taken into account when interpretation these data. For example, there are concerns that the early changes in the synergist ablation model are partly related to edema and nonmuscle cellular infiltration (24–26), and this could feasibly influence muscle wet weight and total DNA content; it could also alter the total RNA and mRNA profile. The reported very high correlation (*r* = 0.93) between rRNA content and growth (16) reflected the regression analysis using the group mean values (rather than raw data), thus elevating the strength of the linear association. A similar problem arises when the relationship between anabolic genes and growth is plotted across, rather than with, independent muscle types (27).

In healthy young men, increases in key ribosome biogenesis regulatory factors were observed after acute resistance exercise both before and after training, with increases in muscle rRNA observed after training (15). However, no change in total muscle RNA was observed after resistance training, and the reported 4-fold increase in 18S seems implausible given it only approximates total RNA, while ∼90% of total RNA is rRNA. Recent work from this laboratory has provided evidence that deficits in both MPS and RNA synthesis underlie blunted hypertrophic responses to exercise with aging in humans (11). Here, RNA was measured from freeze-dried muscle mass. RNA content increased with resistance training only in young individuals. Nevertheless, no study has presented conclusive evidence that ribosomal biogenesis is a prerequisite for skeletal muscle hypertrophy. Indeed, in the current study, we observed that inhibition of ribosomal biogenesis did not prevent early activation of anabolic signaling factors (*i.e.*, mTOR, p70 S6K1, AKT). Furthermore, inhibition of rRNA synthesis did not prevent IGF-1-mediated increases in myotube diameter and protein accretion. Thus, myotube hypertrophy *in vitro* could occur despite inhibition of rRNA synthesis.

Our initial experiments confirmed effective inhibition of Pol I activity occurred after treatment with CX, both with and without the addition of IGF-1, as assessed by measurement of pre-rRNA expression. There was also some evidence of inhibition of Pol II–dependent transcription with CX treatment because c-Myc was suppressed after 4 h treatment. However, other genes measured in the study were unaffected by CX treatment, suggesting that CX was not generally targeting Pol II and that c-Myc was probably indirectly influenced as a result of Pol I inhibition. However, we acknowledge that use of a single pharmacologic agent to inhibit ribosome biogenesis is a limitation of our study, and that additional pharmacologic compounds targeting Pol I and/or gene knockdown of regulatory components would support the major findings of this study. Gene expression analysis revealed that IGF-1 caused up-regulation of several subunits of Pol I, highlighting one mechanism linking early activation of IGF-1-induced factors and increases in translational capacity. Increases in POLR1A, POLR1B, and POLR1C expression were also observed by 24 h with CX treatment alone, possibly indicating attempts to increases Pol I abundance in the face of inhibition of Pol I–dependent transcriptional activity. Conversely, POLR1E mRNA expression was not increased after Pol I inhibition at 24 h and was significantly reduced in CX + IGF-1–treated myotubes. POLR1E encodes a Pol I elongation factor that in yeast has been shown to be important for regulating the recruitment and release of TIF1A during rDNA transcription (28, 29). Of the Pol I subunits measured in this study, only POLR1E does not have a counterpart in the Pol II or Pol III complexes (30), and they thus may be regulated in a different manner than the other subunits. As with the Pol I subunits, the expression of ribosomal protein genes showed differential regulation by IGF-1 and with Pol I inhibition. For example, while RPS5 and RPL13A were largely unaffected by IGF-1 or CX treatment, both RPL32 and RPS19 were markedly up-regulated by IGF-1, and by 24 h, these increases were completely prevented by Pol I inhibition.

Increased expression of ribosomal protein genes has been associated with increased cellular growth, and there is evidence that c-Myc may up-regulate ribosomal proteins (9, 31) *via* the mTOR pathway (32). The present observations imply that ribosomal protein genes can in fact be regulated by a variety of upstream factors, potentially reflecting c-Myc-dependent and -independent regulatory pathways. Examination of Pol I regulatory factors showed that IGF-1 induced early (4 h) increases in c-Myc as well as UBF, TIF1A, and TAF1A, all of which play a crucial role in Pol I preinitiation complex assembly and rDNA transcription. During the early stages of Pol I–dependent transcription, binding of UBF to the rDNA promotor allows recruitment of the SL1 complex, of which TAF1A is a part (1, 2). TIF1A interacts directly with Pol I and is essential for mediating the interaction of Pol I and SL1 to form the preinitiation complex at the rDNA promoter (1, 2). Inhibition of Pol I did not prevent early up-regulation of these factors, and while at 24 h the up-regulation of UBF, TIF1A, and TAF1A by IGF-1 was not affected by CX, c-Myc was significantly reduced compared to IGF-1 alone. The patterns of change in expression of UBF, TIF1A, and TAF1A suggest they may be regulated by a common upstream factor, but because c-Myc expression appeared to be suppressed at this time point, it could potentially occur independently of c-Myc.

It has been reported that serum-induced growth of human myotubes was completely prevented when rRNA synthesis was inhibited with CX (14). In a separate study, Pol I inhibition blocked increases in protein content and MPS with high serum stimulation in C2C12 myotubes, where it was demonstrated that mTOR signaling plays a direct role in rDNA transcription in the early phases of muscle hypertrophy (18). In agreement with a positive role for ribosomal biogenesis for *in vivo* muscle mass regulation, work from our laboratory has demonstrated that age-related deficits in anabolic responses to exercise include impaired ribosomal biogenesis (11). Thus, the findings of the present study, where increases in both myotube diameter and protein accretion with IGF-1 treatment were not prevented by CX, were unexpected. While it is unclear why such differences were observed between the various cell-based studies, this could reflect the differing growth stimulus used; for example, use of 20% serum provides a battery of hormones and growth factors, triggering multiple pathways to growth in comparison with our use of only IGF-1. Thus, using IGF-1 alone, we were evaluating solely IGF-1-specific effects on muscle growth, rather than high serum (fetal bovine serum), which contains a wide composition and variable quantity of growth factors (including embryonic), hormones, and nutrients. Additionally, other cell- or experimental-related differences (*e.g.*, the dose of CX used—previous studies used a 2-fold higher dose than in the present study—cell species, donor characteristics, and stage of differentiation at which experiments were performed) could feasibly have accounted for the differences observed.

It is well established that the mTORC1 signaling pathway is an important regulator of translational efficiency (*i.e.*, protein synthesis rates) and translational capacity in skeletal muscle (3). Activation of mTORC1 stimulates formation of the Pol I transcription complex *via* UBF phosphorylation (2), but it has also been linked to the control of ribosome biogenesis through Pol II– and Pol III–dependent mechanisms (3). Conversely, mTOR-independent mechanisms have been described for growth-related changes in regulation of ribosome biogenesis, with rapamycin-insensitive changes in UBF phosphorylation and c-Myc observed after electrical stimulation in rats (33). In the present study, blocking rRNA synthesis did not prevent the acute IGF-1-mediated increases in anabolic signaling components (phospho-total mTOR, p70 S6K1, AKT), indicating the early increases in translational efficiency with IGF-1 did not require *de novo* ribosome biogenesis. Evaluation of Pol I regulatory factors showed that IGF-1 robustly increased phosphorylation of UBF and total protein levels of TIF1A and c-Myc at 4 h. These findings are consistent with previous studies showing stimulation of ribosomal regulatory factors with growth stimuli (13, 15, 34), and they confirm that anabolic stimuli play a crucial role in increasing translational capacity through Pol I–dependent rDNA transcription. More work is required to fully elucidate the specific pathways involved in activation of the factors required for increased protein synthesis.

We also evaluated the impact of inhibition of ribosomal biogenesis on IGF-1-mediated changes in markers of muscle protein breakdown. Inhibition of ubiquitin-dependent protein breakdown is thought to occur in part *via* AKT-mediated phosphorylation of FOXO transcription factors, which leads to down-regulation of E3 ubiquitin ligases muscle atrophy F-box (MAFbx) and MuRF1 (35–37). We found that IGF-1-mediated increases in FOXO3a phosphorylation were not prevented by Pol I inhibition, although MuRF1 protein levels were unchanged across treatments. This corresponded with a reduction in levels of ubiquitinated protein in both the IGF-1 and CX + IGF-1 groups. Although protein ubiquitination is required for many biologic processes, these findings might indicate that IGF-1-mediated reduction in protein breakdown was not affected by the inhibition of ribosome biogenesis. FOXO3a has also been implicated in regulation of autophagic-lysosomal catabolic processes, partly *via* transcription of the autophagy protein LC3B (38, 39), whereby conversion of the form LC3I to LC3II is used as a marker of autophagy. IGF-1 treatment resulted in a reduction in the LC3II/I ratio, and this was no different in the presence of CX, although it should be noted that the reduced LC3II/I ratio was due to an increase in LC3I rather than a reduction in LC3II. Overall, these findings suggest that the changes in muscle protein breakdown and autophagy after IGF-1 treatment were not affected by inhibition of rRNA synthesis.

Recent studies have investigated downstream effects of CX in relation to its antiproliferative and apoptotic properties, with one study reporting that CX caused DNA damage and apoptotic cell death in various cancer cell lines (40). However, CX has been reported to exert apoptotic effects specifically on cancer cell and not normal cells *in vivo* (41), suggesting that different cells types show different sensitivities to the compound. Previous work using CX with skeletal muscle cells used similar doses to our study with no reported effects on cell viability (14, 18), and in the present study, 24 h of treatment with CX alone caused no changes in total protein or DNA content, suggesting that the dose and duration of treatment did not cause adverse effects related to cell viability. It has also been demonstrated that CX treatment for 72 h induced autophagy and altered mTOR signaling in osteosarcoma cell lines (42). While in the present study treatment with CX alone did not appear to influence mTOR-related signaling, it should be acknowledged that longer-term treatment with the compound might have induced adverse effects related to mTOR inhibition and autophagy.

In summary, this study demonstrated that growth factor–mediated myotube hypertrophy, as assessed by increases in myotube diameter and protein accretion, does not require activation of rRNA synthesis. While ribosome biogenesis still represents a central component of skeletal muscle mass, the present findings imply that there does not appear to be a prerequisite for *de novo* ribosome synthesis in enabling muscle cell growth in response to IGF-1. It is important to acknowledge the limitations of using an *in vitro* model to study processes related to muscle mass regulation and ribosomal biogenesis. This is similar to synergist ablation models used to study the role of satellite cells or ribosomal biogenesis in muscle growth, which can offer some mechanistic insight but have limited use for understanding physiologic muscle growth *in vivo*. While we focused on myotube hypertrophy after 24 h of IGF-1 treatment, future experiments incorporating longer-term treatments could feasibly identify a point at which suppression of ribosome biogenesis does become limiting for continued myotube growth. Thus, future work should focus on understanding the roles of translational capacity and efficiency in the regulation of muscle hypertrophy, including use of *in vivo* models where the Pol I regulatory components are selectively manipulated to determine the impact on the pathways controlling skeletal muscle growth.

## Supplementary Material

Supplemental Data

## Abbreviations

CX: CX-5461
FOXO3a: forkhead box O 3a
LC3B: light chain 3B
MPS: muscle protein synthesis
mTOR: mammalian target of rapamycin
MuRF1: muscle RING finger 1
Pol I: polymerase I
POLR1A/B/C/E: RNA polymerase 1 subunit A/B/C/E
Rb: retinoblastoma
rDNA: ribosomal DNA
RP: ribosomal protein
rRNA: ribosomal RNA
SL1: selectivity factor 1
TAF1A: TATA-box binding protein associated factor, RNA polymerase I, A
TIF1A: transcription initiation factor 1A
UBF: upstream binding factor
