# Supplementary material for: A dynamic ribosomal biogenesis response is not required for IGF-1–mediated hypertrophy of human primary myotubes
Source: FASEB J. 2017 Aug 3;31(12):5196–207. doi: 10.1096/fj.201700329R (PMC5690393; doi:10.1096/fj.201700329R)
Supplement: Supplemental Data [file supp_31_12_5196__index.html]

A dynamic ribosomal biogenesis response is not required for IGF-1–mediated hypertrophy of human primary myotubes — A dynamic ribosomal biogenesis response is not required for IGF-1–mediated hypertrophy of human primary myotubes — A dynamic ribosomal biogenesis response is not required for IGF-1–mediated hypertrophy of human primary myotubes — Supplemental Data 

# A dynamic ribosomal biogenesis response is not required for IGF-1–mediated hypertrophy of human primary myotubes

## Supplemental Data

- Supplemental Data
