# Supplementary material for: A dynamic ribosomal biogenesis response is not required for IGF-1–mediated hypertrophy of human primary myotubes
Source: FASEB J. 2017 Aug 3;31(12):5196–207. doi: 10.1096/fj.201700329R (PMC5690393; doi:10.1096/fj.201700329R)
Supplement: Supplemental Data [file supp_fj.201700329R_Supplemental_Table1.docx]

| **Gene name** | **Forward primer sequence (5’ to 3’)** | **Reverse primer sequence (5’ to 3’)** |
| --- | --- | --- |
| 45S pre-rRNA | GGTGTTTCCTCGTACCGCA | AAGGCTTTTCTCACCGAGGG |
| 28S rRNA | AGAGGTAAACGGGTGGGGTC | GGGGTCGGGAGGAACGG |
| 5.8S rRNA | CCCGTTTGCTGTCTCGTCTG | CGAGTGATCCACCGCTAAGAG |
| 18S rRNA | GATGGTAGTCGCCGTGCC | GCCTGCTGCCTTCCTTGG |
| POLR1A | CCTCAAGGTATCGCCCAGTC | GGCAACTTCTGTTCTTGGGC |
| POLR1B | TACTGTGCAACTTGGGGGTC | GAGAATCTGCGATGCCTGGA |
| POLR1C | AGTCCAAGGTAAAAAGGTGGC | GGCTTCACTCACCAGCACAT |
| POLR1E | CAGATGTGGAGAGCCGAGAC | AGGTCCCAGAGCACTTTTCC |
| UBF | AAGAAGCCTCCCATGAACGG | CGGCCAGCTTTTTGTAGTGC |
| TIF1A | CATTTTGTGCCTCCCCGAGT | GTATTGGCATGAGAAACCACGG |
| c-MYC | GGTAGTGGAAAACCAGCAGCC | TCTCCTCCTCGTCGCAGTA |
| TAF1A | AGGTTTAGCGCCTGCTCATA | CTGAAATCACTCATACCCGCCT |
| RPL13A | TAAACAGGTACTGCTGGGCCG | CTCGGGAAGGGTTGGTGTTC |
| RPL32 | TACGACCCATCAGCCCTTGC | CATGATGCCGAGAAGGAGATGG |
| RPS5 | ATCATCAACAGTGGTCCCCG | AGATGGCCTGGTTCACACG |
| RPS19 | AAACCCCGTCGTTCCCTTTC | GCTTCCCGGACTTTTTGAGG |

**Supplementary Table 1. Primer sequences used in the study.**
